# siRNA targeting decoy receptor 3 enhances the sensitivity of gastric carcinoma cells to 5-fluorouracil

**DOI:** 10.3892/etm.2012.606

**Published:** 2012-06-08

**Authors:** XIAO-TAO XU, ZE-ZHANG TAO, QI-BIN SONG, YI YAO, PENG RUAN

**Affiliations:** Department of Oncology, Renmin Hospital, Wuhan University, Hubei 430060, P.R. China

**Keywords:** decoy receptor 3, RNA interference, gastric cancer, 5-fluorouracil, sensitivity

## Abstract

In order to investigate the effects of RNA interference of decoy receptor 3 (DcR3) on the sensitivity of gastric cancer cells to 5-fluorouracil (5-FU) and the relevant mechanisms, siRNA against DcR3 was transfected into the gastric cancer cell line AGS. AGS cells were treated with different doses of 5-FU or for different time periods. The sensitivity of AGS cells to 5-FU was determined. The cell survival rate was detected by MTT assay. The apoptotic rate was determined by DAPI staining, and the expression of related proteins were detected by western blot analysis. The results showed that the cell survival rate was significanlty decreased in the knockdown group compared to the control group at different doses of 5-FU (P<0.01). After different time periods of treatment with 5-FU, the cell survival rate in the knockdown group was significantly decreased compared to the control group, respectively (P<0.01). The apoptotic rate of AGS cells in the knockdown group was increased along with the increasing dose of siRNA. The siRNA against DcR3 enhanced the expression of Fas, FasL, caspase-3 and caspase-8. In conclusion, knockdown of DcR3 by RNA interference enhances apoptosis and inhibits the growth of gastric cancer cells. Downregulation of DcR3 enhances the sensitivity of gastric cancer cells to 5-FU and increased the expression of Fas, FasL and caspase-3/8.

## Introduction

Decoy receptor 3 (DcR3) is a member of the tumor necrosis factor superfamily. It is structurally similar to functional membranous receptors, but it cannot transduce signals. It competes with functional receptors for ligands, thus blocking ligand-induced apoptosis and promoting tumor cell growth. It has been demonstrated that DcR3 expression relates to the clinical stage, lymphatic metastasis and prognosis of gastric carcinoma (1,2). In addition, DcR3 expression is an indicator of malignancy of gastric carcinoma and may guide gastric carcinoma treatment. However, the role of DcR3 in gastric carcinoma cell sensitivity to chemotherapy has not been reported. In order to investigate the effect of DcR3 on gastric carcinoma cell sensitivity to chemotherapy and the possible mechanism, we observed the effect of DcR3 interference on sensitivity to 5-fluorouracil (5-FU) through transfection of various concentrations of DcR3 siRNA into gastric carcinoma AGS cells and explored the possible mechanism in view of FasL/Fas.

## Materials and methods

### Cell lines and antibodies

Human gastric carcinoma strain AGS was preserved in our laboratory. DMEM medium containing 10% fetal bovine serum was purchased from Gibco-BRL (USA). Goat anti-human DcR3, Fas, FasL, p-caspase-8 and p-caspase-3 antibodies were purchased from Santa Cruz Biotechnology, Inc. (USA). Horseradish peroxidase (HRP)-conjugated rabbit anti-goat secondary antibody was purchased from Boster Biotechnology (Wuhan, China).

### Construction of DcR3 siRNA sequences and transfection of AGS cells

DcR3 cDNA sequence was retrieved from GenBank, and three siRNA sequences were designed according to the literature (3), namely, S1 sense, 5′-UCGACUUUGUGGCUUUC CA-3′ and antisense, 5′-UGGAAAGCCACAAAGUCGA-3′; S2 sense, 5′-ACACCACCTACCCCTGGC-3′ and antisense, 5′-CTA CCTGGAGCGCTGCCGC-3′; S3 sense, 5′-GCCAGGCUCUU CCUCCCAU-3′ and antisense, 5′-AUGGGAGGAAGAGCCU GGC-3′. The negative control Sn sense, 5′-GCCCGCUUUCCC UCAGCAUdTdT-3′ and antisense, 5′-AUGCUGAGGGAAAG CGGGC-3′ were also designed and synthesized. All sequences were synthesized by Takara Biotechnology, Co., Ltd. (Dalian, China) and verified by sequencing. The sequences (100 nM) were transfected into AGS cells using Oligofectamine™ (Invitrogen, USA). There were six groups: the blank group (Con-B), the void vector group (Con-A), the S1 transfection group (S1), the S2 transfection group (S2), the S3 transfection group (S3) and the Sn transfection group (Sn). The blank group was transfected with PBS and the void vector group with void vectors of the same concentration. The other treatments were the same among all of the groups. siRNA with the highest interference efficiency was used in the following assay.

### MTT assay of cell survival

The MTT assay kit was purchased from Takara Biotechnology, Co., Ltd. The cultured cells were inoculated into 96-well plates (1×10^3^ cells/well). Upon cell adherence after 1 day of culture, 20 μl of MTT solution (5 g/l) was added to each well and incubated for 4 h at 37°C. In the control group, 20 μl of PBS was added to each well. After removal of the supernatants, 150 μl of DMSO was added and vortexed thoroughly for 10 min, followed by determination of absorbance (A) at 490 nm in a microplate reader.

$$\text{Cell survival rate}={\text{A}}_{\left(\text{treatment group}\right)}÷{\text{A}}_{\left(\text{control group}\right)}\times 100\%.$$

### Western blot analysis of protein expression

Cells were lysed for 30 min in RIPA lysis buffer and cell lysates were centrifuged for 15 min at low temperature. Total cell protein was measured in the supernatants. Protein (50 μg) was added into 2X loading buffer, denatured at 100°C for 5 min, and separated by SDS-PAGE. Then proteins were transferred onto a nitrocellulose filter. The filter was incubated with specific primary and secondary antibodies, followed by enhanced chemiluminescence (ECL) (Boster Biological Technology, Ltd., China) and autoradiography. The resultant autoradiograms were subjected to grayscale analysis with BandScan software.

### Apoptosis detection

Upon ∼85% cell confluence, cells were digested, centrifuged and prepared into single-cell suspension. Clean coverslips were placed into a 6-well plate, and into each well 2 ml DMEM medium and the cell suspension (5×10^4^/well) were added. Cells were cultured overnight at 37°C in air containing 5% CO_2_. DAPI dye (0.2 ml/well) was added to stain the adhering cells. After 5 min of incubation at room temperature, DAPI dye was removed and cells were washed twice with PBS (5 min/wash). Cells were observed using laser confocal microscopy. Apoptotic cells exhibited dense, darkly stained nuclei or broken bits. The apoptosis rate was calculated by counting the number of apoptotic cells in a total of 100 cells.

### Statistical analysis

Data are expressed as the mean ± SD and a two-sided t-test was performed using SPSS16.0 software. P<0.05 was considered to indicate statistical significance.

## Results

### siRNA effect on DcR3 protein expression

**Figure 1:**
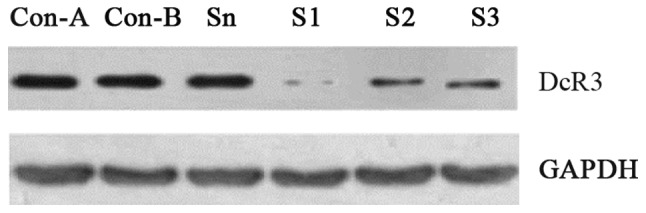
siRNA transfection significantly downregulated DcR3 protein expression, particularly in the S1 group.

Western blot analysis showed that DcR3 protein expression levels were similarly high in the Con-A, Con-B and Sn groups (P>0.05). siRNA transfection significantly (P<0.01) downregulated DcR3 protein expression, particularly in the S1 group (91.3% reduction vs. the control group) (Fig. 1). The results demonstrated that siRNA transfection specifically silenced the DcR3 gene and suppressed DcR3 protein expression in AGS cells. S1 interference was used in the following assays.

### Effect of DcR3 interference on the survival of 5-FU-treated AGS cells

**Figure 2:**
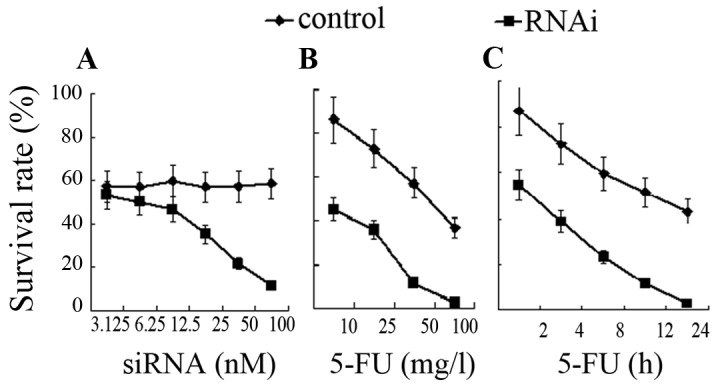
The cell survival rate decreased significantly in the interference group after various durations of 5-FU treatment when compared to the control group.

AGS cells were transfected with various concentrations of S1 siRNA (3.125, 6.25, 12.5, 25, 50 and 100 nM) or PBS. Then cells were treated with 5-FU (final concentration, 50 mg/l) for 24 h. The MTT assay showed that the cell survival rate tended to decrease with increasing concentrations of S1 siRNA. The cell survival rate decreased significantly at 25 nM when compared to the control group (35.3±3.2 vs. 56.8±7.4%) (P<0.05). The cell survival rate decreased significantly at 50 nM (21.6±2.6%) and at 100 nM (11.6±1.5%) when compared to the control group (57.2±7.6 and 58.4±8.3%, respectively) (P<0.01, Fig. 2A). AGS cells were then transfected with 100 nM siRNA (interference group) or with isovolumetric PBS (control group). Cells were treated with various concentrations of 5-FU (10, 25, 50 and 100 mg/l) for 24 h and cell survival rates were determined. The results showed that after 5-FU treatment, cell survival rate decreased significantly in the interference group when compared to the control group (P<0.05), and tended to decrease with increasing 5-FU concentrations (Fig. 2B). In addition, AGS cells were transfected with 100 nM siRNA (interference group) or with isovolumetric PBS (control group). Both groups were treated with 100 mg/l 5-FU for various time periods (2, 4, 8, 12 and 24 h). The results showed that cell survival rate decreased significantly in the interference group after various durations of 5-FU treatment when compared to the control group (P<0.01) (Fig. 2C).

### Effect of DcR3 interference on the apoptosis of 5-FU-treated AGS cells

**Figure 3:**
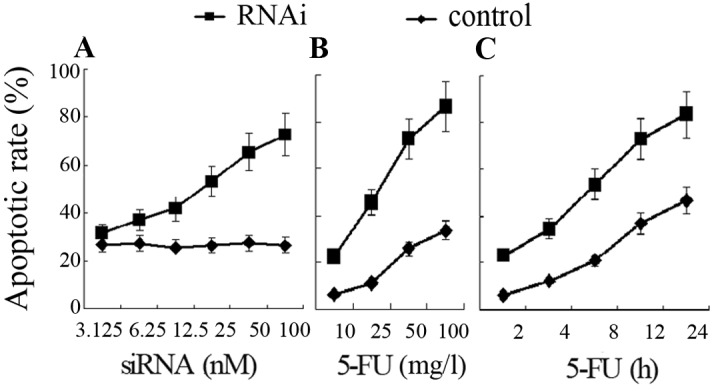
The apoptosis rate increased significantly in the interference group, (A) with increasing concentrations of DcR3 siRNA, (B) after treatment with different doses of 5-FU and (C) after various durations of 5-FU treatment.

DAPI staining showed that the apoptotic rate of AGS cells tended to increase with increasing concentrations of DcR3 siRNA. The apoptotic rate increased significantly at 12.5 nM when compared to the control group (41.6±5.2 vs. 25.6±3.3%) (P<0.05). With increasing concentrations of DcR3 siRNA, the apoptotic rate increased significantly when compared to the control group (P<0.01) (Fig. 3A). After 5-FU treatment (10, 25, 50 and 100 mg/l), the apoptotic rate increased significantly in the interference group (22.6, 45.7, 72.9 and 86.7%, respectively) when compared to the control group (6.2, 11.3, 25.8 and 33.6%, respectively) (P<0.01) (Fig. 3B). After various durations of 5-FU treatment (2, 4, 8, 12 and 24 h), the apoptotic rate increased significantly in the interference group when compared to the control group (P<0.01, Fig. 3C).

### Effect of DcR3 interference on Fas and FasL protein expression

**Figure 4:**
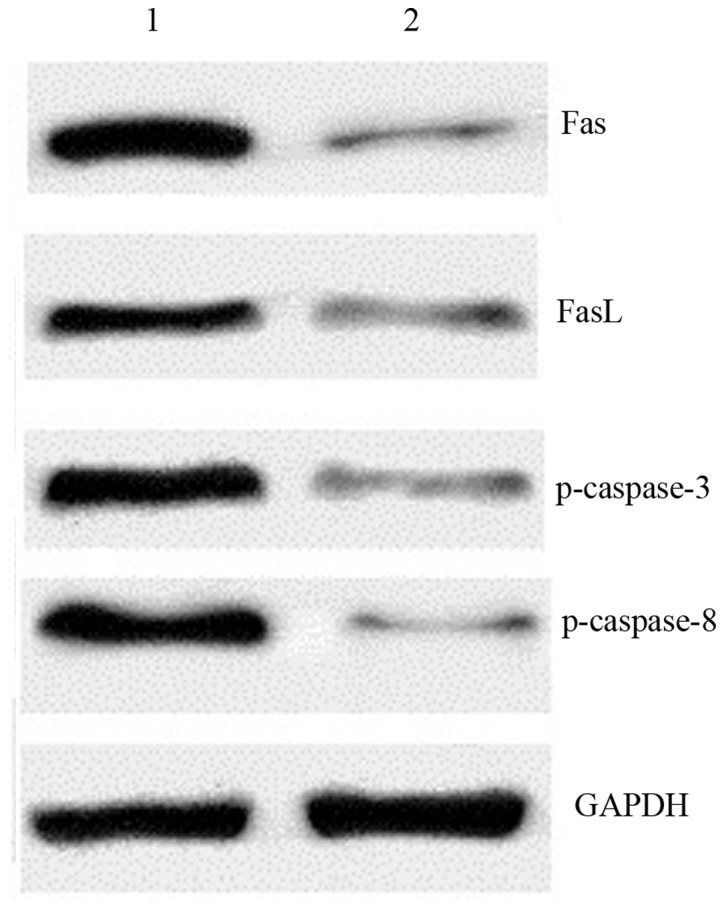
After 24 h of treatment with 100 mg/l 5-FU, western blot analysis results showed that Fas and FasL expression increased significantly in the interference group and that phosphorylated (p)-caspase-3 and p-caspase-8 expression also increased. Lane 1, interference group; lane 2, control group.

AGS cells were transfected with 100 nM DcR3 siRNA (interference group) or isovolumetric PBS (control group). After 24 h of treatment with 100 mg/l 5-FU, western blot analysis of Fas, FasL, phosphorylated caspase-3 and phosphorylated caspase-8 was performed in both groups. The results showed that Fas and FasL expression increased significantly in the interference group (0.86±0.18, 0.72±0.15) when compared to the control group (0.15±0.08, 0.21±0.09) (P<0.01), and that phosphorylated caspase-3 and phosphorylated caspase-8 expression increased significantly in the interference group (0.82±0.13, 0.85±0.16) when compared to the control group (0.16±0.05, 0.12±0.02) (P<0.01) (Fig. 4).

## Discussion

DcR3 encoding gene M68 is mapped to human chromosome 20q13.3 and the sequence comprises 3 exons. DcR3 protein comprises 271 amino acids and its molecular weight is 35 kDa. It is a soluble secretory protein without a transmembranous sequence. DcR3 binds to FasL (4), LIGHT (5) and TLIA3 (6) and suppresses their apoptosis-inducing activity. Hence, DcR3 is able to suppress apoptosis, promote cell growth and regulate immunity, thus playing an important role in human tumorigenesis, tumor malignancy and prognosis (7). In addition, DcR3 is associated with tumor cell sensitivity to 5-FU-based chemotherapy (8).

Since DcR3 is associated with the incidence and development of tumors, and disease prognosis, the role of the suppression of DcR3 activity in tumor therapy and prognosis has become a focus of research. In recent years, RNA interference technology has been widely used to silence DcR3 expression and assess the role of DcR3 in the incidence, development and treatment of tumors. Interference of DcR3 expression was suggested to suppress the growth (9) and promote the apoptosis of colon cancer cells. It has been demonstrated that DcR3 expression levels are correlated with lung cancer cell sensitivity to chemotherapy and that DcR3 interference may enhance lung cancer cell sensitivity to chemotherapy (10).

In the present study, specific DcR3 siRNA sequences were transfected into gastric carcinoma AGS cells to block DcR3 expression. DcR3 siRNA sequence that showed the highest interference efficiency (up to 90%) and high specificity was screened (Fig. 1). In order to investigate the effect of DcR3 interference on gastric carcinoma cell sensitivity to 5-FU, we determined the cell survival rate of 5-FU-treated gastric carcinoma cells transfected with various concentrations of siRNA. The results showed that the cell survival rate tended to decrease with increasing concentrations of siRNA, and that cell survival rates were maintained between 50–60% when cells were not transfected. At 100 nM DcR3 siRNA, cell survival rate decreased to ∼10%. This suggests that DcR3 interference may enhance gastric carcinoma cell sensitivity to 5-FU (Fig. 2A). In addition, various concentrations of 5-FU significantly decreased the survival rate of transfected AGS cells and the median lethal concentration (LC_50_) of 5-FU decreased to under 10 mg/l in transfected cells. In contrast, LC_50_ of 5-FU was approximately 75 mg/l in AGS cells which were not transfected (Fig. 2B). Moreover, DcR3 interference reduced the median lethal time (LT_50_) of 5-FU from 24 to 2 h (Fig. 2C). These results demonstrated that DcR3 interference enhanced AGS cell sensitivity to 5-FU. 5-FU is an important chemotherapeutic agent for gastric carcinoma, and it suppresses and kills tumor cells through inducing apoptosis. Fig. 3 demonstrates that DcR3 siRNA promoted 5-FU-induced gastric carcinoma apoptosis in a time- and concentration-dependent manner. These findings demonstrate that DcR3 interference enhanced AGS cell sensitivity to 5-FU through suppression of cell growth and promotion of apoptosis.

The molecular mechanism by which DcR3 affects gastric carcinoma cell sensitivity to 5-FU may involve multiple signaling pathways. It was demonstrated that activating the PI3K/Akt/NF-κB signaling pathway downregulated DCR3 expression and increased FasL-induced apoptosis (11). DcR3 may induce cell proliferation through activating the MAPK pathway (12). The DcR3-TL1A signaling pathway may suppress cytokine-induced cell proliferation (13). Fas and FasL are key apoptosis-inducing factors, and they are molecular targets for tumor therapy (14). In the present study, the molecular mechanism by which DcR3 promotes gastric carcinoma cell sensitivity to 5-FU was investigated in view of Fas and FasL. The results demonstrated that DcR3 interference upregulated Fas and FasL protein expression, while activating the apoptosis-related proteins caspase-3 and caspase-8. DcR3 and FasL are structurally similar; hence, DcR3 competes with FasL, but it cannot transduce a signal. Consequently, DcR3 suppression reduces competitive FasL suppression. The caspase family is the executor of apoptosis, leading to morphological and biological changes in apoptosis.

In summary, the present study demonstrated that DcR3 interference promoted 5-FU-induced apoptosis, suppressed cell growth and enhanced gastric carcinoma cell sensitivity to 5-FU. In addition, blocking DcR3 expression enhanced the expression of Fas, FasL and caspase-3/8. However, the molecular mechanism of DcR3 is complex, and further investigation is required to assess DcR3 as a molecular target for tumor therapy.
